# Pyoderma gangrenosum with complications of severe trigeminal neuralgia

**DOI:** 10.1016/j.jdcr.2024.09.011

**Published:** 2024-10-01

**Authors:** Tara Ghalambor, Lindsay Ackerman

**Affiliations:** a University of Arizona College of Medicine – Phoenix, Phoenix, Arizona; b Department of Dermatology, Banner University Medical Center, Phoenix, Arizona

**Keywords:** adalimumab, ear, external ear, inflammatory skin diseases, neutrophilic dermatoses, pyoderma gangrenosum, trigeminal neuralgia, tumor necrosis factor (TNF) inhibitors

## Background

Pyoderma gangrenosum (PG) is an inflammatory skin disease characterized by progressive painful skin ulcerations distinguished clinically by pathergy and chronicity, and morphologically with raised undermined borders, often with a purple-gray discoloration along the margin of adjacent skin.^1^ Although not completely understood, the pathogenesis of PG is believed to be multifactorial involving many inflammatory cytokines contributing to neutrophil dysfunction and occasionally found to be associated with systemic inflammatory diseases.^2^ Pain is a major comorbidity associated with PG, albeit limited to the cutaneous site(s) of ulceration. We present a case of a woman presenting with a PG lesion on her right ear and retro-auricular area, with symptoms of severe trigeminal neuropathic pain involving her cheek and ipsilateral nose, mimicking trigeminal neuralgia.

## Case presentation

**Fig 1:**
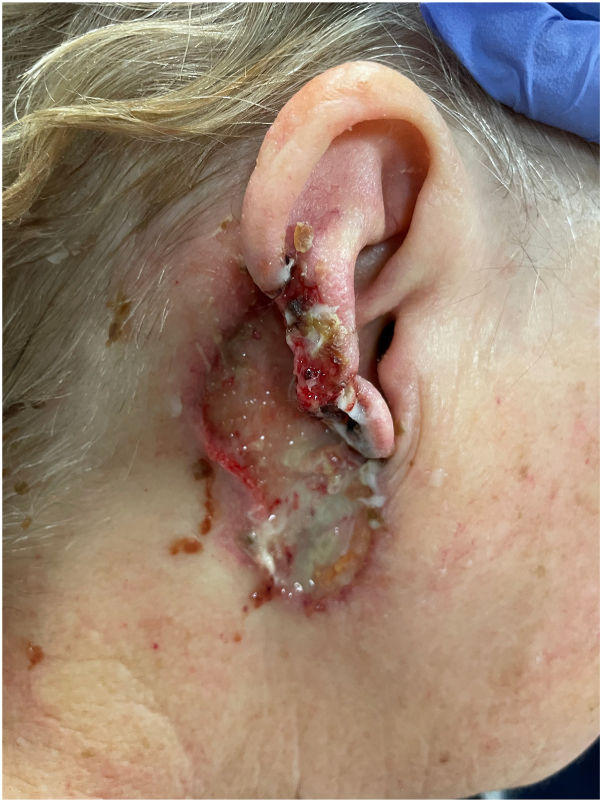
Pyoderma gangrenosum of the auricle and retro-auricular area. Initial hospital presentation showing pustular ulceration under serosanguinous crust of the right auricle and proximal neck at the angle of the mandibular jaw line.

**Fig 2:**
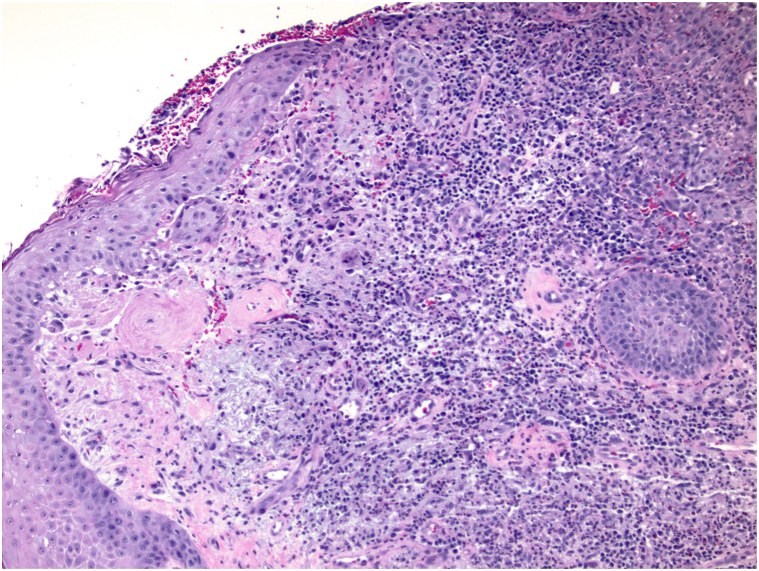
Biopsy of the back of the right ear showing brisk granulomatous and neutrophilic inflammation without vasculitis.

A 69-year-old woman presented to the hospital with a few weeks’ history of severe progressive neuropathic pain of the right medial cheek, right nasal sidewall, and jaw, leading to difficulty with eating. Upon interview and examination, it was notable that she had a 2-year history of a chronic ulceration of yet to be determined etiology, of the right auricle and retro-auricular area extending to the angle of the mandible. The ulcer was characterized by a lack of intact auricular cartilage, crusted serosanguinous debris with underlying purulence covering the ulceration, which upon gentle debridement revealed undermined borders along the margins (Fig 1). The patient’s presentation to the hospital was because of severe new onset pain wherein there was *no* visible cutaneous pathology, on the right medial cheek and right nasal sidewall, consistent with a V2 distribution of the trigeminal nerve. The severity of pain in these areas was debilitating, leading to an inability to sleep, and subsequent weight loss of 10 pounds from the inability to eat. Notably, 1 month before this presentation, the patient had visited an outpatient dermatology office where a 1.3 cm × 0.7 cm × 0.3 cm excisional biopsy of the right scapha and shave biopsy of an ulcerated region of the superior right side of the neck was performed due to concerns for a basal cell carcinoma. Histology of both sites revealed ruptured epidermoid cysts with neutrophilic and granulomatous inflammation. Two weeks later, with a worsening wound, the patient was reevaluated, and another shave biopsy was performed on the back of the ear with differential diagnosis of PG. Biopsy results showed brisk granulomatous inflammation, with sheets of neutrophils without vasculitis (Fig 2). Significant medical history included bilateral seronegative arthritis of patellofemoral joints and hips, Charcot-Marie-Tooth deformity, muscular dystrophy, and previous history of lower extremity ulcers that had healed with antibiotics and hyperbaric oxygen therapy.

**Fig 3:**
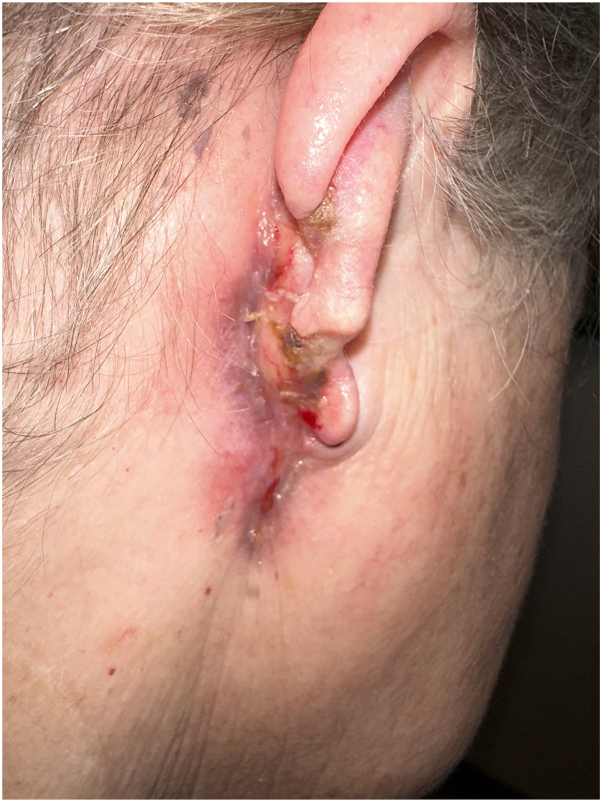
Pyoderma gangrenosum of the auricle and retro-auricular area. Presentation after 1 month of adalimumab showing significant contraction and re-epithelialization of ulceration with no new areas of involvement.

**Fig 4:**
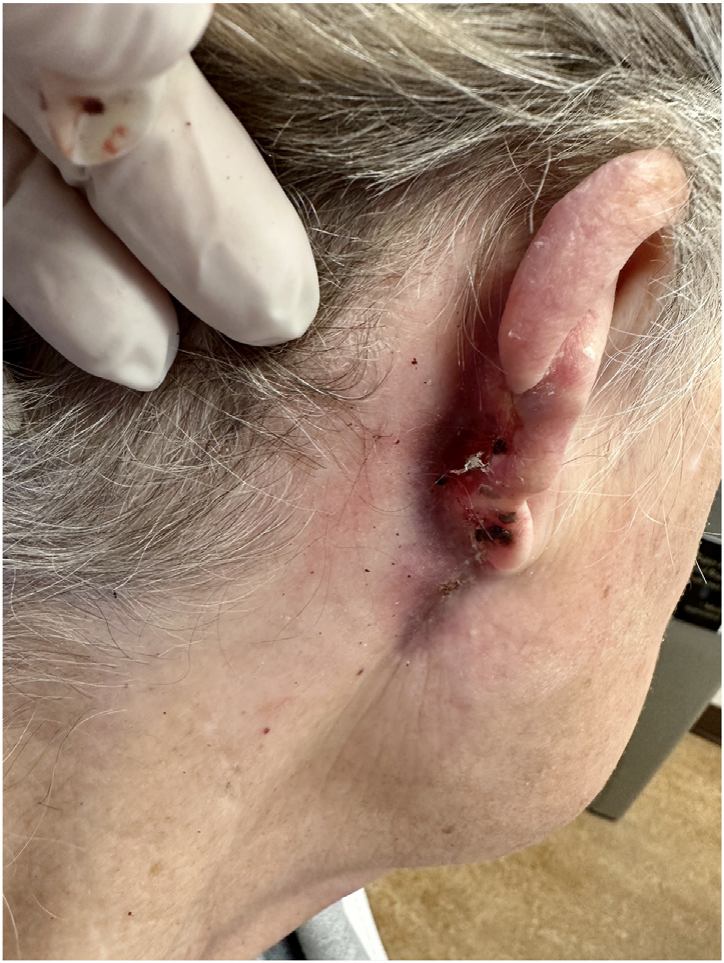
Pyoderma gangrenosum of the auricle and retro-auricular area. Presentation at 3 months showing complete healing of the lesion.

Upon admission, the patient received empiric treatment for neuropathic pain with methylprednisolone, amitriptyline, and carbamazepine. A secondary pseudomonas infection was identified on wound culture from the purulence underlying the serosanguinous debris on the ulceration. The ulceration was treated with gentle debridement, followed by administration of intravenous cefepime and topical silver sulfadiazine cream. The previous biopsy results, obtained from the outpatient dermatology office, were reviewed by a pathologist who was provided with the patient’s clinical history. Based on the clinical presentation, chronicity of the ulceration, presence of a ruptured cyst with secondary granulomatous inflammation, and the history of 2 traumatic procedures before substantial clinical exacerbation, there was sufficient clinicopathologic evidence to support a diagnosis of PG. Concurrently, a work-up for systemic lupus erythematosus was negative, as were QuantiFERON and hepatitis serologies. With a diagnosis of PG, and a secondary severe trigeminal neuralgia, adalimumab was initiated. Within 1 month, there was near complete re-epithelialization of the PG, and as the wound began to promptly improve, there was concurrent prompt improvement in neuropathic pain (Fig 3). This rapid improvement in the skin lesion and trigeminal pain led to successful discontinuation of amitriptyline and carbamazepine, both minimally effective in granting pain control. As the wound has remained re-epithelialized, the patient has maintained a complete eradication of neuropathic pain, in the area that was otherwise refractory to neuromodulation (Fig 4).

## Discussion

We present a case of symptoms of severe trigeminal neuralgia as a manifestation of retro-auricular and auricular PG. Although the exact pathogenesis of trigeminal neuralgia remains poorly understood, it is believed to involve vascular compression of the trigeminal nerve with damage to the myelin sheath, leading to severe and recalcitrant pain along the trigeminal distribution.^3^ Although the location of the PG lesion does not overlie the origin of the trigeminal nerve, we suspect the local inflammatory process of this ulceration could cause this specific pain distribution. Reports of PG lesions involving the auricle and its surroundings have been noted, although without concomitant trigeminal pathology leading to debilitating neuropathy.^4^, ^5^, ^6^, ^7^ Of note, there has been one other case of facial PG that has led to involvement of the facial nerve leading to keratopathy because of paralysis of the eyelid and cicatricial ectropion.^8^

Because of the rarity of this disease and lack of specific histologic or serologic markers, PG is a challenging diagnosis that is often delayed and can lead to extensive tissue damage, as was the case with our patient.^9^ To help with this, a Delphi consensus of international experts have validated diagnostic criteria for classic ulcerative PG, consisting of 1 major criterion of biopsy of the ulcer edge demonstrating neutrophilic infiltrate and 8 minor criterion, 4 of which need to be met to conclude PG.^10^ Although the biopsy of this patient also included granulomatous infiltrate, the neutrophilic infiltrate in the biopsy, in addition to her history of inflammatory arthritis, physical examination findings, pathergy, and response to immunosuppressive medications support this diagnosis. As PG is a diagnosis of exclusion, it is important to consider a biopsy of the lesion to rule out other etiologies such as malignancy or vasculitides, but to remain careful that physical trauma to the site may be subject to pathergy.^2^ Successful PG treatment for extensive disease often ultimately requires systemic immunomodulation with tumor necrosis factor-alpha inhibitors, making clinical familiarity with PG, its potential subsequent sequelae, as well as potential underlying comorbid pathologies, of importance.^11^

## Conflicts of interest

None disclosed.
